# Association of long-term use of non-steroidal anti-inflammatory drugs with knee osteoarthritis: a prospective multi-cohort study over 4-to-5 years

**DOI:** 10.1038/s41598-024-56665-3

**Published:** 2024-03-19

**Authors:** Zubeyir Salis, Amanda Sainsbury

**Affiliations:** 1 Division of Rheumatology, Geneva University Hospital and Faculty of Medicine, University of Geneva, HUG Av. de Beau-Séjour 26, 1206 Geneva, Switzerland; 2 Centre for Big Data Research in Health, The University of New South Wales, Kensington, NSW Australia; 3 School of Human Sciences, The University of Western Australia, Perth, WA Australia

**Keywords:** Knee osteoarthritis, Non-steroidal anti-inflammatory drug, NSAID, Osteoarthritis, Pain management

## Abstract

This study examines the long-term impact of non-steroidal anti-inflammatory drugs (NSAIDs) on the progression of symptoms and structural deterioration of the joint in knee osteoarthritis. The study analyzes data from 4197 participants (8394 knees) across the Osteoarthritis Initiative (OAI), Multicenter Osteoarthritis Study (MOST), and Cohort Hip and Cohort Knee (CHECK) over 4-to-5 years. Adjustments were made for major covariates. We focussed on binary outcomes to assess the presence or absence of significant changes. We found that, relative to non-users, individuals using NSAIDs long-term were significantly more likely to experience aggravated symptoms exceeding the minimally clinically important difference, specifically, pain (OR: 2.04, 95% CI: 1.66–2.49), disability (OR: 2.21, 95% CI: 1.74–2.80), and stiffness (OR: 1.58, 95% CI: 1.29–1.93). Long-term users also faced a higher probability than non-users of having total knee replacement (OR: 3.13, 95% CI: 2.08–4.70), although no significant difference between long-term users and non-users was observed for structural deterioration in the knee joint (OR: 1.25, 95% CI: 0.94–1.65). While acknowledging the limitations of this study due to its observational design and the potential for bidirectional causality, these findings suggest that long-term NSAID use could accelerate the progression to total knee replacement by markedly exacerbating symptoms.

## Introduction

Non-steroidal anti-inflammatory drugs (NSAIDs) are widely used for their pain-relieving effects across a variety of ailments, including osteoarthritis (OA)^1^. However, they are associated with significant risks, contributing to 30% of hospital admissions for adverse drug reactions^2^. These risks include serious gastrointestinal complications^3^, heightened risk of cardiovascular disease^4^, and renal failure^5^. More specifically, research indicates that 13–15% of NSAID users experience upper gastrointestinal adverse effects^6^, and a quarter of peptic ulcer cases may result from NSAID use alone^7^. Moreover, NSAIDs are associated with a 25% increased risk of cardiovascular events^8^. Consequently, international guidelines discourage NSAID use in certain individuals, particularly those with comorbidities or cardiovascular disease, and recommend short-term use only^9–12^.

Despite the above-mentioned guidelines, long-term use of NSAIDs remains prevalent. A recent study revealed that patients with hip and knee OA were prescribed NSAIDs for an average duration of approximately 16 months over three years of observation^13^. Moreover, the prevalence of long-term use of NSAIDs is on the rise. In 2010, 29 million (12.1% of) adults in the United States of America (USA) reported long-term use of NSAIDs (defined as a usage duration of over three months)^14^, representing a 41% increase since 2005. Patients with OA account for a significant portion of these users, with nearly 65% of patients with OA and chronic low back pain in the USA prescribed NSAIDs for chronic pain management^15^.

Despite the prevalence of long-term NSAID use for pain management, existing research provides an incomplete picture of their effects on knee osteoarthritis (KOA) symptoms and structural changes. Prior systematic reviews and meta-analyses have predominantly focused on the short-term effects of NSAIDs, covering periods of use ranging from 2 to 54 weeks (12 months)^16–24^. These findings suggest that NSAIDs may alleviate or improve KOA symptoms during this short-term period. However, questions regarding the potential long-term impact, extending beyond 54 weeks, remain unanswered by these previous meta-analyses^16–24^. A limited number of studies^25–27^ covered in these previous meta-analyses^16–24^ have explored longer-term use, specifically, durations between 80 and 144 months, but their findings have been inconsistent^25–27^. Thus, the impact of long-term use of NSAIDs on symptoms and structural changes in KOA is unclear.

These previous meta-analyses were limited in their ability to explore the long-term effects of NSAID use on KOA symptoms and structural changes, not only due to the limited number of long-term studies available for inclusion but also due to their reliance on aggregated data. Aggregated data meta-analyses summarize information from a number of studies, with those studies often employing varied statistical methods, varied exposure measures, and varied outcome measures. This approach can obscure the true association between exposure and outcomes, compromising the accuracy of the conclusions^28^. Conversely, analyzing individual-level data from multi-cohorts within a single, unified analysis can more accurately depict the relationship between exposure and outcome measures. In light of this, we used individual-level data from three major OA cohorts (the Osteoarthritis Initiative (OAI), the Multicenter Osteoarthritis Study (MOST), and the Cohort Hip and Cohort Knee (CHECK) study) to examine the association of long-term use of NSAIDs over 4-to-5 years and progression of symptoms and structural changes in KOA, as well as total knee replacement (TKR).

## Methods

### Study design, setting, and participants

We obtained publicly available data from three independent cohorts: the OAI^29^; the MOST^30^; and the CHECK study^31^. These cohorts primarily comprised participants with KOA or at risk of developing it, and the CHECK study additionally included some participants with or at risk of hip OA. The OAI enrolled participants at four clinical centers in the USA between 2004 and 2006. The MOST enrolled participants at two clinical centers in the USA between 2003 and 2005. The CHECK enrolled participants at ten clinical centers in the Netherlands between 2002 and 2005. There were 4,796, 3,026, and 1,002 adults in the OAI, MOST, and CHECK study cohorts, respectively. This study did not require ethical approval as it relies on already published data and does not involve a new collection of data from, or direct interaction with, human subjects. The OAI, MOST, and CHECK studies had their own appropriate ethical approvals at the time of original data collection.

We used symptomatic data, specifically, pain, disability, and stiffness, and radiographic (X-ray) data, from baseline and at the 4-year follow-up from the OAI study, and from baseline and at the 5-year follow-up from the MOST and CHECK studies. The OAI study had limited radiographic data at any other time points beyond 4 years. The MOST and CHECK studies did not have radiographic data at 4 years, and that is why the 5-year data were used.

### Exposure

The exposure in our study was the long-term use of NSAIDs. We determined NSAID use in the OAI and MOST cohorts using the medication inventory method^32^. This involves participants bringing all their current medications to a study visit, where researchers document the names and other relevant details of their medications. This approach provides an accurate assessment of medication use, minimizing recall bias and allowing for drug verification. In OAI and MOST, NSAID use was defined as any prescribed use of NSAIDs (including COX2 inhibitors) in any route and form (including oral capsules) within the last 30 days at the time of the assessment. In the CHECK cohort, we determined NSAID use (including COX2 inhibitors) based on the information reported during the clinical interview. Participants were defined as NSAID users if they answered ‘yes’ to the question of whether they were using ibuprofen, diclofenac, naproxen, celecoxib, or rofecoxib for their complaints of hip or knee at the time of the assessment.

A participant was classified as a long-term NSAID user if they met the criteria for NSAID usage, as defined above, at baseline and all follow-up visits. The follow-up time points occurred annually over 4 years in OAI (years 1 to 4) and over 5 years in CHECK (years 1–5), and every 2.5 years over 5 years in MOST (years 2.5 and 5). Based on literature indicating that many patients remain on NSAID treatment for durations averaging between 16 ± 12 months^13^ and 20 ± 16 months^33^, we assumed continuous NSAID use between visits. A participant was classified as a non-user of NSAID if they did not meet the NSAID usage criteria at any visit, whether at baseline or during follow-up.

### Outcomes

We investigated the association of NSAID use with KOA outcomes across two domains over a 4-to-5-year follow-up: symptoms and structural changes. Specifically, we analyzed five binary outcomes: three pertaining to symptoms of KOA and two to structural changes. While our primary focus was on binary outcomes, we have also included average changes in symptom severity from baseline to the 4-to-5-year follow-up as secondary, continuous outcomes for supplementary insight, as detailed in the “Statistical analyses” section below.

#### Symptoms of KOA

The Western Ontario and McMaster Universities Arthritis Index (WOMAC) was used to assess changes in KOA symptoms, specifically pain, disability, and stiffness. WOMAC scores ranged from 0 to 20 for pain, 0 to 68 for disability, and 0 to 8 for stiffness. Higher scores indicate worse symptoms. The minimal clinically important difference (MCID) for symptom worsening was defined as ≥ 6.4 normalized units (NU, 0–100 scale) for pain, ≥ 10.3 NU for disability, and ≥ 2.9 NU stiffness^34^. We examined three binary outcomes related to KOA symptoms: worsening pain, worsening disability, and worsening stiffness, all of which were above their respective MCID values.

#### Structural changes in KOA

Structural changes were assessed by KOA severity grade as assessed by radiography and TKR. We used Kellgren/Lawrence (K/L) grades^35^, which range from 0 to 4, to evaluate KOA severity. Higher K/L grades indicate more severe KOA. We considered two binary outcomes for structural changes: worsening in radiographic KOA severity grade, and incidence of TKR. The outcome of worsening in radiographic KOA severity grade was defined as a knee with an increase of ≥ 1 K/L grades at the 4-to-5-year follow-up compared to baseline. We did not define an increase from K/L grade 0 at baseline to K/L grade 1 at 4-to-5-year follow-up as a worsening in radiographic KOA because K/L grade 1 is not uniformly considered as KOA^35^. In addition, we excluded knees with a baseline K/L grade of 4, the highest possible grade, from the analysis of radiographic KOA worsening, as no further deterioration could be detected for these knees. The outcome of incidence of TKR was defined as a knee undergoing TKR at any time point between baseline and the 4-to-5-year follow-up.

### Inclusion criteria

**Figure 1:**
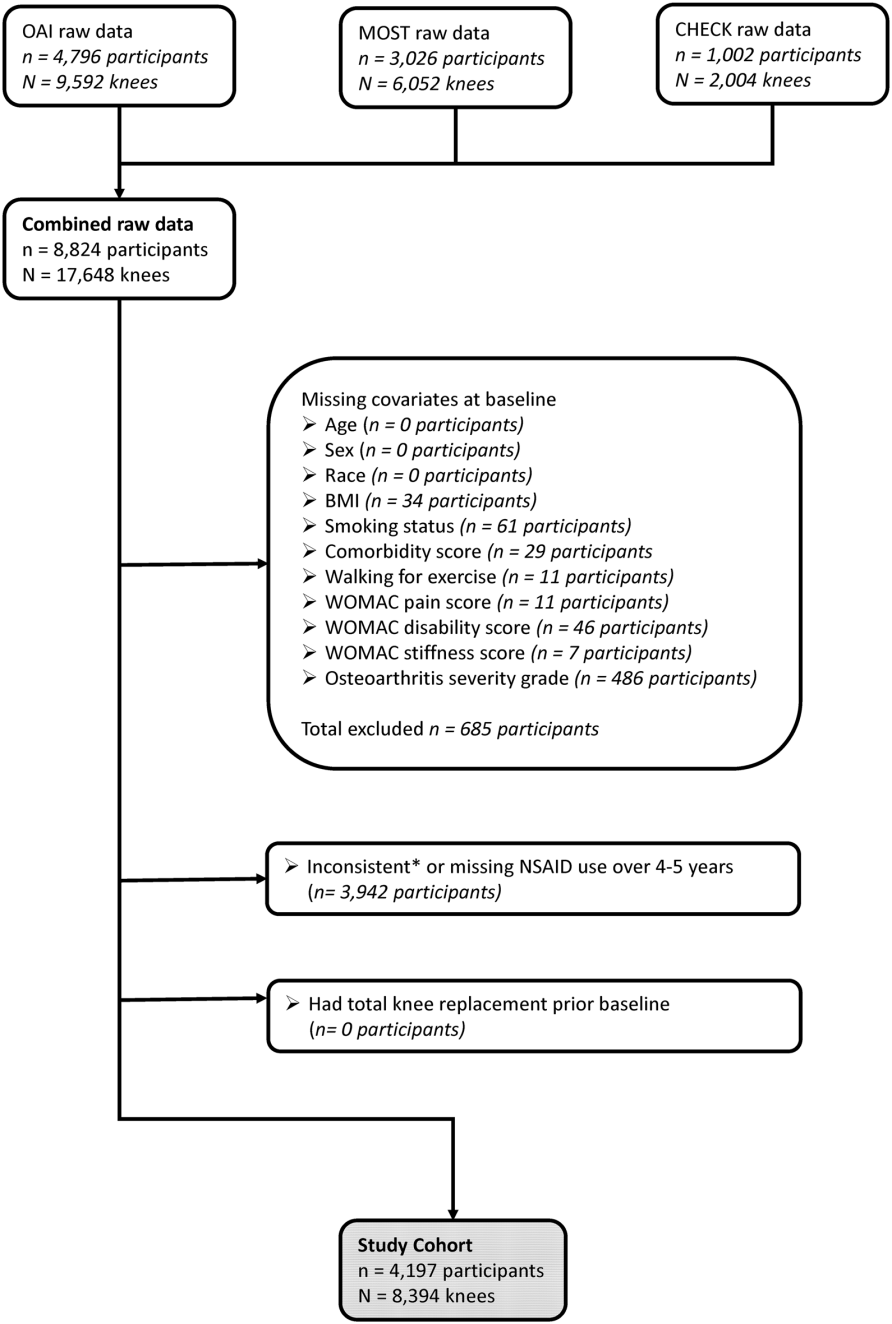
Selection of participants. * NSAID use reported in the last 30 days at the time of the assessment at baseline and annually over 4 years in OAI; in the last 30 days at the time of the assessment at baseline and every 2.5 years over 5 years in MOST, at the time of the assessment at baseline and annually over 5 years in CHECK. BMI, body mass index; CHECK, Cohort of Hip and Cohort of Knee; MOST, multicenter osteoarthritis study; NSAID, non-steroidal anti-inflammatory drug; OAI, Osteoarthritis Initiative study; WOMAC, Western Ontario and McMaster Universities Arthritis Index.

We only included participants with complete data at baseline for the covariates used in our analyses. Participants also needed to have enough data available to be classified as long-term NSAID users or NSAID non-users to be included in our analyses (Fig. 1).

### Statistical analyses

We performed analyses of individual-level data from specific OA cohorts within a single, unified analysis to estimate the associations between the long-term use of NSAIDs and the above-mentioned outcomes over 4-to-5 years. As a statistical model, we used generalized estimating equations with a logistic link function (i.e., logistic regression), clustering the left and right knee of each participant. The analyses were adjusted for the following covariates: sex, race, and baseline values of age, Body Mass Index (BMI), smoking status, comorbidity score, walking for leisure or activity, WOMAC pain score, WOMAC disability score, WOMAC stiffness score, OA severity by K/L grade, and study cohort (OAI, MOST, and CHECK). These covariates were selected prior to the analyses due to their associations with KOA, which was found in previous research. In addition, we reported the characteristics of long-term NSAID users and NSAID non-users at baseline compared by calculating absolute standardized mean differences (SMDs) between them. An SMD > 0.10 indicates a possible imbalance for that characteristic between groups^36^.

As mentioned above, we assessed the presence or absence of significant symptoms and structural changes using binary, primary outcomes. In addition to these binary, primary outcomes, we also evaluated the severity of symptoms over time through secondary, continuous outcomes, as measured by the WOMAC scores, specifically WOMAC pain, WOMAC disability, and WOMAC stiffness scores. These scores were reported as average differences between baseline and the 4-to-5-year follow-up, both in adjusted and unadjusted forms. We employed linear mixed models for the adjusted estimations of average differences for WOMAC scores, using the participant as a random effect since we included the left and right knee of each participant.

We used STATA/BE 17.0 for Windows (64-bit × 86–64) for our analyses. We adjusted the significance level for multiple testing (i.e., Bonferroni adjustment); therefore, we set our threshold for statistical significance as a two-tailed P-value of less than 0.01 (0.05/5 primary outcomes).

#### Missing data analyses

At baseline, we ensured a complete dataset by excluding any entries with missing data, as detailed in Fig. 1. However, at the 4-to-5-year follow-up, we encountered instances of missing outcome data. We performed missing data analyses to assess the potential impact of missing outcome data at the 4-to-5-year follow-up on our estimates. Our tests showed that the missing data for those outcomes were missing at non-random (MNAR), so we did not perform multiple imputations. Instead, we used best-case and worst-case scenarios by replacing the missing outcomes with positive outcomes (e.g., no worsening pain that was above the MCID value at 4-to-5-year follow-up) and negative outcomes (e.g., worsening pain that was above the MCID value at 4-to-5-year follow-up), respectively. Missing data analysis was not performed for the incidence of TKR due to challenges in distinguishing between the actual absence of TKR and the absence of data about TKR within the datasets used.

#### Sensitivity analysis

As a sensitivity analysis, we matched participants in the long-term NSAID users group with participants in the NSAID non-users group, employing 1:1 nearest-neighbor matching with propensity scores. The propensity scores were calculated based on the variables that we used for adjusting the estimates for our main analyses. These variables were sex, race, and baseline values of age, BMI, smoking status, comorbidity score, walking for leisure or activity, WOMAC pain score, WOMAC disability score, WOMAC stiffness score, osteoarthritis severity as indicated by K/L grade, and study cohort (OAI, MOST, or CHECK). After propensity score matching, we compared the characteristics of participants in the long-term NSAID user and NSAID non-user groups at baseline by calculating absolute standardized mean differences (SMDs) between groups to determine if there were significant differences between the groups. As in the main analysis, we considered an SMD > 0.10 to indicate a possible imbalance for that characteristic between groups. We then compared the outcomes over 4-to-5 years between the groups using the same statistical methods as in the main analyses.

## Results

### Participants

Our study included 4,197 participants, of whom 435 were long-term NSAID users, and 3762 were non-users. Long-term NSAID users and NSAID non-users were similar in age (range 45–79 years), race, smoking status, comorbidity scores, and walking status for leisure or exercise. However, long-term NSAID users were more likely than non-users to be female, heavier, and also had higher scores for symptoms of pain, disability, and stiffness, and were more likely to have radiographic KOA (i.e., K/L grade ≥ 2) (Table 1).

**Table 1:** Baseline characteristics of participants, by long-term use of non-steroidal anti-inflammatory drugs (NSAIDs).

Characteristics	Non-users	Users*	Standardized mean difference
	Participants n = 3762 (89.64)	Participants n = 435 (10.36)	
Age, years [range: min to max]	61.07 ± 8.75 [45 to 79]	61.40 ± 8.35 [45 to 79]	− 3.76
Sex			33.93
Male	1602 (42.58)	116 (26.67)	
Female	2160 (57.42)	319 (73.33)	
Race			6.84
Non-white	530 (14.09)	72 (16.55)	
White	3232 (85.91)	363 (83.45)	
BMI, kg/m^2^ [range: min to max]	28.12 ± 4.83 [16.72 to 54.08]	30.45 ± 5.79 [18.60 to 50.47]	− 43.60
Smoking status			7.02
Never	2604 (69.22)	287 (65.98)	
Former	986 (26.21)	125 (28.74)	
Current	172 (4.57)	23 (5.29)	
Comorbidity score			2.43
0	2679 (71.21)	307 (70.57)	
1	598 (15.90)	82 (18.85)	
2 or more	485 (12.89)	46 (10.57)	
Walking for leisure or exercise			2.04
Never	466 (12.39)	51 (11.72)	
Seldom/Sometimes/Often	3296 (87.61)	384 (88.28)	
Study cohort			68.69
OAI	2600 (69.11)	194 (44.60)	
MOST	739 (19.64)	219 (50.34)	
CHECK	423 (11.24)	22 (5.06)	

	Knees N = 7524 (89.6)	Knees N = 870 (10.4)	
WOMAC pain score [range: min to max]	11.03 ± 14.85 [0 to 90]	18.82 ± 18.70 [0 to 100]	− 46.10
WOMAC disability score [range: min to max]	11.47 ± 14.71 [0 to 90]	22.12 ± 18.81 [0 to 94]	− 63.09
WOMAC stiffness score [range: min to max]	17.90 ± 19.32 [0 to 100]	27.50 ± 21.59 [0 to 100]	− 46.86
Osteoarthritis severity by K/L grade			− 38.71
0: None	3392 (45.08)	291 (33.45)	
1: Doubtful	1518 (20.18)	132 (15.17)	
2: Mild	1593 (21.17)	201 (23.10)	
3: Moderate	816 (10.85)	173 (19.89)	
4: Severe	205 (2.72)	73 (8.39)	

Data are presented as mean ± standard deviation or count (percentage). * NSAID use reported in the last 30 days at the time of the assessment at baseline and annually over 4 years in OAI; in the last 30 days at the time of the assessment at baseline and every 2.5 years over 5 years in MOST, at the time of the assessment at baseline and annually over 5 years in CHECK.

BMI, body mass index; CHECK, Cohort Hip and Cohort Knee; CI, confidence intervals; K/L, Kellgren/Lawrence; MOST, Multicenter Osteoarthritis Study; OAI, osteoarthritis initiative; SD, standard deviation; WOMAC, Western Ontario and McMaster Universities Arthritis Index.

### Outcomes

#### Symptoms of KOA

At the 4-to-5-year follow-up, compared to non-users, long-term NSAID users had an increase in adjusted mean pain score of 3.95 (95% confidence interval [CI]: 2.68–5.21), adjusted mean disability score of 5.37 (95% CI: 3.98–6.76), and adjusted mean stiffness score of 4.34 (95% CI: 2.73–5.94) (Table 2).

**Table 2:** Association of long-term use of non-steroidal anti-inflammatory drugs (NSAIDs) with symptoms and structural changes in knee osteoarthritis over 4-to-5 years.

Outcome measures	Non-users N = 7524 (knees)	Users^a^ N = 870 (knees)
WOMAC pain score		
Unadjusted mean at baseline (SD)	11.03 ± 14.85	18.82 ± 18.70
Unadjusted mean at 4-to-5-year follow-up (SD)	10.06 ± 14.56	18.96 ± 18.89
Absolute change, mean (95% CI)	− 0.97 (− 1.29 to − 0.66) (N = 7504)	0.14 (− 1.07 to 1.35) (N = 870)
Adjusted mean (95% CI)	− 1.27 (− 1.61 to − 0.93)	2.68 (1.49 to 3.87)
Adjusted mean difference (95% CI)	0.00 (ref.)	3.95 (2.68 to 5.21)
Incidence of worsening pain that was above the MCID value^b^	1292 (17.22) (N = 7504)	264 (30.34) (N = 870)
Odds ratio (95% CI)	1.00 (ref.)	2.04 (1.66 to 2.49)
WOMAC disability score		
Unadjusted mean at baseline (SD)	11.47 ± 14.71	22.12 ± 18.81
Unadjusted mean at 4-to-5-year follow-up (SD)	10.61 ± 14.80	22.21 ± 18.97
Absolute change, mean (95% CI)	− 0.76 (− 1.05 to − 0.47) (N = 7469)	0.34 (0.78 to 1.45) (N = 856)
Adjusted mean (95% CI)	− 1.20 (− 1.56 to − 0.84)	4.17 (2.87 to 5.48)
Adjusted mean difference (95% CI)	0.00 (ref.)	5.37 (3.98 to 6.76)
Incidence of worsening disability that was above the MCID value^b^	901 (12.06) (N = 7469)	194 (22.67) (N = 856)
Odds ratio (95% CI)	1.00 (ref.)	2.21 (1.74 to 2.80)
WOMAC stiffness		
Unadjusted mean at baseline (SD)	17.90 ± 19.32	27.50 ± 21.59
Unadjusted mean at 4-to-5-year follow-up (SD)	16.43 ± 19.47	27.50 ± 21.88
Absolute change, mean (95% CI)	− 1.46 (− 1.89 to − 1.03) (N = 7,497)	0.00 (− 1.48 to 1.48) (N = 870)
Adjusted mean (95% CI)	− 1.76 (− 2.23 to − 1.29)	2.58 (1.08 to 4.07)
Adjusted mean difference (95% CI)	0.00 (ref.)	4.34 (2.73 to 5.94)
Incidence of worsening stiffness that was above the MCID value^b^	1941 (25.89) (N = 7497)	309 (35.52) (N = 870)
Odds ratio (95% CI)	1.00 (ref.)	1.58 (1.29 to 1.93)
Worsening in radiographic knee osteoarthritis severity grade^c^		
Incidence, N (%)	977 (14.85) (N = 6,580)	180 (26.09) (N = 690)
Odds ratio (95% CI)	1.00 (ref.)	1.43 (1.15 to 1.77)
Incidence of total knee replacement		
Incidence, N (%)	104 (1.38) (N = 7524)	79 (9.08) (N = 870)
Odds ratio (95% CI)	1.00 (ref.)	3.13 (2.08 to 4.70)

Data are presented as mean ± standard deviation or count (percentage).

BMI, body mass index; CHECK, Cohort Hip and Cohort Knee; CI, confidence intervals; K/L, Kellgren/Lawrence; MCID, Minimum Clinically Important Difference; MOST, Multicenter Osteoarthritis Study; OAI: Osteoarthritis Initiative; SD, standard deviation; WOMAC, Western Ontario and McMaster Universities Arthritis Index. P-values were not reported (they were less than 0.01).

^a^NSAID use reported in the last 30 days at the time of the assessment at baseline and annually over 4 years in OAI; in the last 30 days at the time of the assessment at baseline and every 2.5 years over 5 years in MOST, at the time of the assessment at baseline and annually over 5 years in CHECK.

^b^The minimal clinically important differences: pain ≥ 6.4 normalized units (NU), disability ≥ 10.3 NU, stiffness ≥ 2.9 NU.

^c^K/L grade increase of ≥ 1 at 4-to-5-year follow-up from baseline (increase from K/L grade 0 at baseline to K/L grade 1 at 4-to-5-year follow-up not included). Adjusted for sex, race, and baseline values of age, BMI, smoking status, comorbidity score, walking for leisure or activity, WOMAC pain score, WOMAC disability score, WOMAC stiffness score, osteoarthritis severity by K/L grade, and study cohort (OAI, MOST, and CHECK).

At the 4-to-5-year follow-up, 1,292 (17.22% of) non-users and 264 (30.34% of) long-term NSAID users had worsening pain scores that were above the MCID value. The odds ratio for worsening pain scores that were above the MCID value was 2.04 (95% CI: 1.66–2.49). The increased odds of worsening symptom scores were also observed for the disability and stiffness scores. At the 4-to-5-year follow-up, 901 (12.06%) non-users and 194 (22.67% of) long-term NSAID users had worsening disability scores that were above the MCID value, and 1,941 (25.89% of) non-users and 309 (35.52% of) long-term NSAID users had worsening stiffness scores that were above the MCID value. The odds ratio for worsening disability scores that were above the MCID value was 2.21 (95% CI: 1.74–2.80), and for stiffness was 1.58 (95% CI: 1.29–1.93) (Table 2).

#### Structural changes in KOA

At the 4-to-5-year follow-up, long-term NSAID users had significantly increased odds of worsening in radiographic KOA severity grade (OR: 1.43, 95% CI: 1.15–1.77), and markedly increased odds of having TKR (OR: 3.13, 95% CI: 2.08–4.70), compared to non-users (Table 2).

### Missing data analyses

The missing data analysis (Table 3) to assess the potential impact of missing outcomes on our estimates also showed that NSAID users had increased odds of the outcomes, with the exception of the outcome of worsening in radiographic KOA severity grade in the worst-case scenario, for which the association did not reach statistical significance (P value 0.027) (Table 3).

**Table 3:** Missing data analyses of the potential impact of missing data on the estimations.

Outcome measures^a^	Worst case	Best case
Symptoms		
Incidence of worsening pain that was above the MCID value^b^		
Odds ratio (95% CI)	2.05 (1.68 to 2.51)	1.99 (1.63 to 2.44)
Incidence of worsening disability that was above the MCID value^b^		
Odds ratio (95% CI)	2.21 (1.74 to 2.80)	2.11 (1.67 to 2.66)
Incidence of worsening stiffness that was above the MCID value^b^		
Odds ratio (95% CI)	1.60 (1.31 to 1.95)	1.55 (1.27 to 1.89)
Structural changes		
Worsening in radiographic knee osteoarthritis severity grade^a^		
Odds ratio (95% CI)	1.27 (1.03 to 1.58)*	1.41 (1.15 to 1.72)

Data are presented as mean ± standard deviation or count (percentage).

^a^No missing data for the incidence of total knee replacement outcome. BMI: body mass index; WOMAC: Western Ontario and McMaster Universities Arthritis Index. Adjusted for baseline values of age, sex, race, BMI, smoking status, comorbidity score, walking for leisure or activity, WOMAC pain score, WOMAC disability score, WOMAC stiffness score, and Kellgren-Lawrence (K-L) grade of the knee. *P-value: 0.027, all the other P-values are less than 0.01.

^b^The minimal clinically important differences: pain ≥ 6.4 normalized units (NU), disability ≥ 10.3 NU, stiffness ≥ 2.9 NU.

### Sensitivity analysis

**Figure 2:**
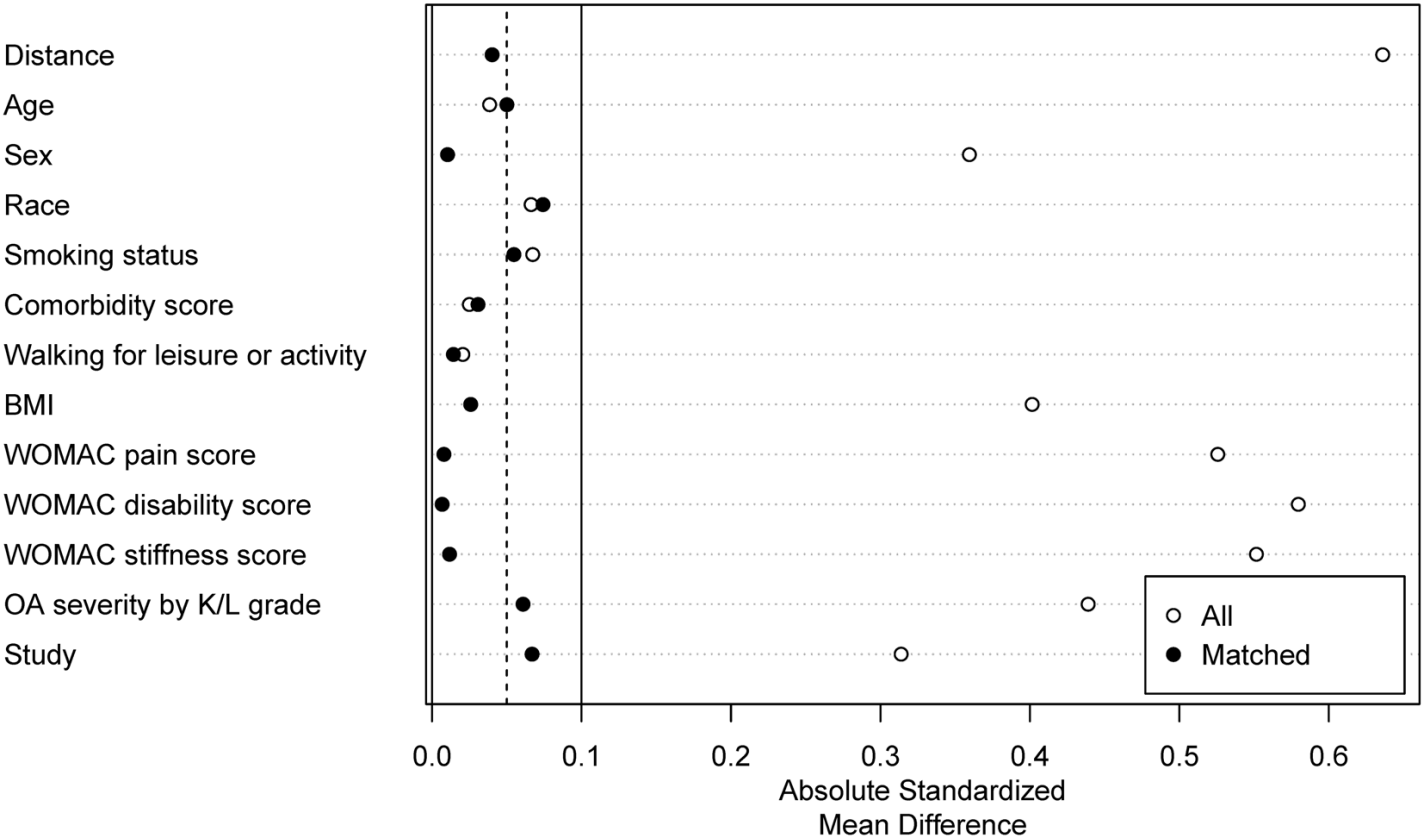
Summary of balance for matched data. Here, ‘distance’ quantifies the level of similarity or difference in key characteristics among the subjects in our study, offering insight into the extent to which individuals are comparable or distinct based on the variables selected for analysis. BMI, body mass index; K/L, Kellgren/Lawrence; OA, osteoarthritis; WOMAC, Western Ontario and McMaster Universities Arthritis Index.

In the sensitivity analysis, in which we used propensity score matching, the matched data were well balanced between the long-term NSAID users group and the non-users group (Fig. 2). All the estimates were similar or the same as in the main analysis, with the exception of worsening in radiographic KOA severity grade over the follow-up period (Table 4), where the odds ratio for this outcome did not achieve statistical significance (OR: 1.25, 95% CI: 0.94–1.65, P value: 0.12).

**Table 4:** Association of long-term use of non-steroidal anti-inflammatory drugs (NSAIDs) with symptoms and structural changes in knee osteoarthritis over 4-to-5 years using 1:1 propensity score matching (PSM).

Outcome measures	Non-users N = 870 (knees)	Users^a^ N = 870 (knees)
WOMAC pain score		
Unadjusted mean at baseline (SD)	19.55 ∓ 19.28	18.82 ∓ 18.70
Unadjusted mean at 4-to-5-year follow-up (SD)	16.18 ∓ 18.42	18.96 ∓ 18.89
Absolute change, mean (95% CI)	− 3.33 (− 4.46 to − 2.18) (N = 866)	0.14 (− 1.07 to 1.35) (N = 870)
Adjusted mean (95% CI)	− 3.23 (− 4.38 to − 2.08)	0.05 (− 1.16 to 1.26)
Adjusted mean difference (95% CI)	0.00 (ref.)	3.28 (1.60 to 4.96)
Incidence of worsening pain that was above the MCID value^b^	157 (18.13) (N = 866)	264 (30.34) (N = 870)
Odds ratio (95% CI)	1.00 (ref.)	2.05 (1.56 to 2.70)
WOMAC disability score		
Unadjusted mean at baseline (SD)	21.84 ∓ 19.77	22.12 ∓ 18.81
Unadjusted mean at 4-to-5-year follow-up (SD)	17.95 ∓ 18.81	22.21 ∓ 18.97
Absolute change, mean (95% CI)	− 3.57 (− 4.62 to − 2.52) (N = 856)	0.34 (0.78 to 1.45) (N = 856)
Adjusted mean (95% CI)	− 3.43 (− 4.67 to − 2.19)	0.19 (− 1.09 to 1.48)
Adjusted mean difference (95% CI)	0.00 (ref.)	3.62 (1.83 to 5.41)
Incidence of worsening disability that was above the MCID value^b^	112 (13.08) (N = 856)	194 (22.66) (N = 856)
Odds ratio (95% CI)	1.00 (ref.)	1.93 (1.37 to 2.72)
WOMAC stiffness		
Unadjusted mean at baseline (SD)	28.23 ∓ 23.07	27.50 ∓ 21.59
Unadjusted mean at 4-to-5-year follow-up (SD)	23.57 ∓ 23.15	27.50 ∓ 21.88
Absolute change, mean (95% CI)	− 4.61 (− 6.11 to − 3.11) (N = 863)	0.00 (− 1.48 to 1.48) (N = 870)
Adjusted mean (95% CI)	− 0.36 (− 0.49 to − 0.23)	− 0.01 (− 0.13 to 0.11)
Adjusted mean difference (95% CI)	0.00 (ref.)	0.35 (0.18 to 0.52)
Incidence of worsening stiffness that was above the MCID value^b^	234 (27.11) (N = 863)	309 (35.52) (N = 870)
Odds ratio (95% CI)	1.00 (ref.)	1.57 (1.20 to 2.05)
Worsening in radiographic knee osteoarthritis severity grade^c^		
Incidence, N (%)	164 (22.84) (N = 718)	180 (26.09) (N = 690)
Odds ratio (95% CI)	1.00 (ref.)	1.25 (0.94 to 1.65)
Incidence of total knee replacement		
Incidence, N (%)	39 (4.5) (N = 870)	79 (9.08) (N = 870)
Odds ratio (95% CI)	1.00 (ref.)	2.26 (1.30 to 3.92)

Data are presented as mean ± standard deviation or count (percentage).

P-values were not reported (less than 0.01, except for the worsening in radiographic knee osteoarthritis severity grade where the P value was 0.12 for this outcome).

BMI, body mass index; CHECK, Cohort Hip and Cohort Knee; CI, confidence intervals; K/L, Kellgren/Lawrence; MCID, Minimum Clinically Important Difference; MOST, Multicenter Osteoarthritis Study; OAI, osteoarthritis initiative; SD, standard deviation; WOMAC, Western Ontario and McMaster Universities Arthritis Index.

^a^NSAID use reported in the last 30 days at the time of the assessment at baseline and annually over 4 years in OAI; in the last 30 days at the time of the assessment at baseline and every 2.5 years over 5 years in MOST, at the time of the assessment at baseline and annually over 5 years in CHECK.

^b^The minimal clinically important differences: pain ≥ 6.4 normalized units (NU), disability ≥ 10.3 NU, stiffness ≥ 2.9 NU.

^c^K/L grade increase of ≥ 1 at 4-to-5-year follow-up from baseline (increase from K/L grade 0 at baseline to K/L grade 1 at 4-to-5-year follow-up not included). Adjusted for sex, race, and baseline values of age, BMI, smoking status, comorbidity score, walking for leisure or activity, WOMAC pain score, WOMAC disability score, WOMAC stiffness score, osteoarthritis severity by K/L grade, and study cohort (OAI, MOST, and CHECK).

## Discussion

Our 4-to-5-year longitudinal study revealed that long-term use of NSAIDs is associated with increased odds of experiencing not only a worsening of symptoms of KOA in individuals with or at risk of KOA compared to non-users but also a worsening that exceeds the minimally clinically important difference, as well as an increased incidence of TKR, without any statistically significant difference in structural degradation of the knee joint. Therefore, it appears that long-term NSAID use may accelerate the pathway to TKR by markedly exacerbating symptoms.

Previous reviews of randomized controlled trials and cohort studies showed that long-term use of NSAIDs is associated with serious adverse drug effects, such as gastrointestinal complications^3^, cardiovascular disease^4^, and renal failure^5^. In addition to these well-documented adverse effects, emerging evidence has suggested a potentially detrimental impact of NSAID use on joint health, particularly in terms of cartilage degeneration^37–42^. However, we did not detect any association of long-term use of NSAIDs with structural degradation of the joint. As our study results suggested that long-term NSAID use may increase KOA symptoms and the need for TKR, healthcare providers are advised to carefully consider the implications of long-term NSAID use for individuals with or at risk of KOA and explore alternative management strategies that may improve patient outcomes. Alternative treatments such as patient education, targeted exercise, and weight management, as recommended in the guidelines for managing KOA^43–46^, offer promising avenues for enhancing patient outcomes with minimal risks. Prioritizing these non-pharmacological interventions could enable patients to effectively manage their KOA symptoms without exposing themselves to the potential hazards associated with prolonged NSAID consumption.

Previous systematic reviews and meta-analyses have shown that NSAIDs may provide short-term pain relief and/or improve KOA functions, but the effects of long-term use were unclear^16–24^. Using individual-level participant data, our study suggested that long-term use of NSAIDs over 4-to-5 years may aggravate KOA symptoms. Additionally, studies on the effect of NSAIDs on structural changes in KOA report conflicting results. Some studies, including cohort, in vitro, and animal studies, report beneficial effects or no effect at all^27,47–49^. However, other studies, specifically within the cohort category, indicate negative effects^50–52^. Our study showed that 4-to-5 years of NSAID use may not have detrimental effects on structural changes in knees affected with KOA. Our study extends the findings from a recent systematic review and meta-analysis study that associated NSAID use with an increased risk of knee replacements^53^. However, that study^53^ included only two case–control studies specifically investigating knee replacement, and it reported high heterogeneity in the meta-analysis results. Therefore, their findings were challenging to interpret. However, our analysis, using individual-level participant data, provides a more reliable conclusion that long-term use of NSAIDs is associated with increased odds of having TKR, and that this observation could be due to the experience of increased symptoms compared to NSAID non-users.

A few limitations in our study should be acknowledged. Firstly, most of our participants were white, which may limit the generalizability of our findings to other populations. Secondly, we did not adjust our results for over-the-counter painkillers and opioid use, which may have led to an overestimation of the magnitude of the risks of long-term use of NSAIDs in our results. Thirdly, while we assumed continuous NSAID use between visits based on literature on average duration of use, this assumption is a limitation in our study, as we lacked direct data to confirm or disprove continuous use of NSAID use among participants, and it is possible that participants used NSAIDs intermittently. Similarly, we acknowledge the lack of specific data on NSAID dosage, the duration of NSAID use prior to baseline, and the timing of NSAID prescriptions. As a fourth limitation, although the application of propensity score matching in our sensitivity analysis has improved the reliability of our findings, it is important to recognize that this method cannot eliminate the impact of unmeasured confounders. Moreover, it does not fully resolve the issue of potential bidirectional causality. Specifically, while long-term NSAID use may increase the symptoms of KOA, an increase in KOA symptoms could also lead individuals to increase their use of NSAIDs. In brief, the observational nature of our study limits causal inference. Finally, although no difference was observed in structural degeneration of the knee joint between long-term NSAID users and non-users, unidentified factors may exacerbate symptoms among NSAID users. To overcome these limitations, future research, especially through randomized controlled trials, is vital.

In conclusion, our findings suggest that long-term use of NSAIDs for the management of KOA may lead to worsening symptoms and greater progression to TKR but not structural changes in the knee joint over 4 to 5 years. Healthcare providers should weigh the benefits and risks of NSAID use in patients with KOA and consider non-pharmacological treatments, such as education, exercise, and weight loss, to improve patient outcomes.

## Data Availability

The datasets were derived from sources in the public domain: OAI public use data sets are available through the National Institute of Mental Health Data Archive, MOST public use data sets are available through the NIA Aging Research Biobank, and CHECK public use data sets are available through the https://easy.dans.knaw.nl/ui/home.

## References

[1] Conaghan PG (2012). A turbulent decade for NSAIDs: Update on current concepts of classification, epidemiology, comparative efficacy, and toxicity. Rheumatol. Int..

[2] Davis A, Robson J (2016). The dangers of NSAIDs: Look both ways. Br. J. Gener. Pract..

[3] Tramèr MR (2000). Quantitative estimation of rare adverse events which follow a biological progression: A new model applied to chronic NSAID use. Pain.

[4] Bleumink GS (2003). Nonsteroidal anti-inflammatory drugs and heart failure. Drugs.

[5] Whelton A (1999). Nephrotoxicity of nonsteroidal anti-inflammatory drugs: Physiologic foundations and clinical implications. Am. J. Med..

[6] Pok LSL (2018). Clinical and economic implications of upper gastrointestinal adverse events in Asian rheumatological patients on long-term non-steroidal anti-inflammatory drugs. Int. J. Rheum. Dis..

[7] Kurata JH, Nogawa AN (1997). Meta-analysis of risk factors for peptic ulcer. Nonsteroidal antiinflammatory drugs, *Helicobacter pylori*, and smoking. J. Clin. Gastroenterol..

[8] Martín Arias LH (2019). Cardiovascular risk of nonsteroidal anti-inflammatory drugs and classical and selective cyclooxygenase-2 inhibitors: A meta-analysis of observational studies. J. Clin. Pharmacol..

[9] Panel, B.t.A.G.S.B.C.U.E. (2015). American Geriatrics Society 2015 updated beers criteria for potentially inappropriate medication use in older adults. J. Am. Geriatr. Soc..

[10] Bannuru RR (2019). OARSI guidelines for the non-surgical management of knee, hip, and polyarticular osteoarthritis. Osteoarthr. Cartil..

[11] Kolasinski SL (2020). 2019 American College of Rheumatology/arthritis foundation guideline for the management of osteoarthritis of the hand, hip, and knee. Arthrit. Care Res..

[12] Arden NK (2021). Non-surgical management of knee osteoarthritis: Comparison of ESCEO and OARSI 2019 guidelines. Nat. Rev. Rheumatol..

[13] Silverman SL (2022). Drug utilization, clinical and economic outcomes of patients with osteoarthritis of the hip and/or knee treated with long-term use of traditional NSAIDs, topical NSAIDs, and COX-2 inhibitors. Curr. Med. Res. Opin..

[14] Zhou Y, Boudreau DM, Freedman AN (2014). Trends in the use of aspirin and nonsteroidal anti-inflammatory drugs in the general U.S. population. Pharmacoepidemiol. Drug Saf..

[15] Gore M (2012). Use and costs of prescription medications and alternative treatments in patients with osteoarthritis and chronic low back pain in community-based settings. Pain Pract..

[16] Bjordal JM (2004). Non-steroidal anti-inflammatory drugs, including cyclo-oxygenase-2 inhibitors, in osteoarthritic knee pain: Meta-analysis of randomised placebo controlled trials. BMJ.

[17] Adatia A, Rainsford KD, Kean WF (2012). Osteoarthritis of the knee and hip. Part II: Therapy with ibuprofen and a review of clinical trials. J. Pharm. Pharmacol..

[18] Deeks JJ, Smith LA, Bradley MD (2002). Efficacy, tolerability, and upper gastrointestinal safety of celecoxib for treatment of osteoarthritis and rheumatoid arthritis: Systematic review of randomised controlled trials. BMJ.

[19] da Costa BR (2017). Effectiveness of non-steroidal anti-inflammatory drugs for the treatment of pain in knee and hip osteoarthritis: A network meta-analysis. The Lancet.

[20] da Costa BR (2021). Effectiveness and safety of non-steroidal anti-inflammatory drugs and opioid treatment for knee and hip osteoarthritis: Network meta-analysis. BMJ.

[21] Osani MC (2020). Duration of symptom relief and early trajectory of adverse events for oral nonsteroidal antiinflammatory drugs in knee osteoarthritis: A systematic review and meta-analysis. Arthrit. Care Res. (Hobok.).

[22] Bannuru RR (2015). Comparative effectiveness of pharmacologic interventions for knee osteoarthritis: A systematic review and network meta-analysis. Ann. Intern. Med..

[23] Ton J (2020). PEER umbrella systematic review of systematic reviews: Management of osteoarthritis in primary care. Can. Fam. Phys..

[24] Bjordal JM (2007). Short-term efficacy of pharmacotherapeutic interventions in osteoarthritic knee pain: A meta-analysis of randomised placebo-controlled trials. Eur. J. Pain.

[25] Scott DL (2000). The long-term effects of non-steroidal anti-inflammatory drugs in osteoarthritis of the knee: A randomized placebo-controlled trial. Rheumatol. (Oxf.).

[26] Dieppe P (1993). A two-year, placebo-controlled trial of non-steroidal anti-inflammatory therapy in osteoarthritis of the knee joint. Br. J. Rheumatol..

[27] Lapane KL (2015). Effects of prescription nonsteroidal antiinflammatory drugs on symptoms and disease progression among patients with knee osteoarthritis. Arthrit. Rheumatol..

[28] Tierney JF (2015). Individual participant data (IPD) meta-analyses of randomised controlled trials: Guidance on their use. PLoS Med..

[29] Nevitt, M. C., David T. F. & Gayle, L. The osteoarthritis initiative. In *Protocol for the cohort study. National Institute of Arthritis, Musculoskeletal and Skin Diseases. V 1.1 6.21.06* (2023, accessed 11 Dec 2023). https://nda.nih.gov/static/docs/StudyDesignProtocolAndAppendices.pdf.

[30] Segal NA (2013). The multicenter osteoarthritis study: Opportunities for rehabilitation research. PM and R.

[31] Wesseling J (2014). Cohort profile: Cohort hip and cohort knee (CHECK) study. Int. J. Epidemiol..

[32] Pahor M (1994). Drug data coding and analysis in epidemiologic studies. Eur. J. Epidemiol..

[33] Nissen SE (2016). Cardiovascular safety of celecoxib, naproxen, or ibuprofen for arthritis. N. Engl. J. Med..

[34] Angst F (2002). Minimal clinically important rehabilitation effects in patients with osteoarthritis of the lower extremities. J. Rheumatol..

[35] Kellgren JH, Lawrence JS (1957). Radiological assessment of osteo-arthrosis. Ann. Rheum. Dis..

[36] Schulte PJ, Mascha EJ (2018). Propensity score methods: Theory and practice for anesthesia research. Anesth. Analg..

[37] Abrams GD, Chang W, Dragoo JL (2017). In vitro chondrotoxicity of nonsteroidal anti-inflammatory drugs and opioid medications. Am. J. Sports Med..

[38] Beitzel K (2013). The effect of ketorolac tromethamine, methylprednisolone, and platelet-rich plasma on human chondrocyte and tenocyte viability. Arthrosc. J. Arthrosc. Relat. Surg..

[39] Dogan N (2004). The effects of ketorolac and morphine on articular cartilage and synovium in the rabbit knee joint. Can. J. Physiol. Pharmacol..

[40] Irwin MG (1998). Intra-articular injection of ketorolac in the rat knee joint: Effect on articular cartilage and synovium. Br. J. Anaesth..

[41] Orak MM (2015). Comparison of the effects of chronic intra-articular administration of tenoxicam, diclofenac, and methylprednisolone in healthy rats. Acta Orthop. Traumatol. Turc..

[42] Ozyuvaci H (2004). Intra-articular injection of tenoxicam in rats: Assessment of the local effects on the articular cartilage and synovium. J. Int. Med. Res..

[43] Fernandes L (2013). EULAR recommendations for the non-pharmacological core management of hip and knee osteoarthritis. Ann. Rheum. Dis..

[44] Rausch Osthoff AK (2018). Effects of exercise and physical activity promotion: Meta-analysis informing the 2018 EULAR recommendations for physical activity in people with rheumatoid arthritis, spondyloarthritis and hip/knee osteoarthritis. RMD Open.

[45] Hochberg MC (2012). American College of Rheumatology 2012 recommendations for the use of nonpharmacologic and pharmacologic therapies in osteoarthritis of the hand, hip, and knee. Arthrit. Care Res..

[46] McAlindon TE (2014). OARSI guidelines for the non-surgical management of knee osteoarthritis. Osteoarthr. Cartil..

[47] Mastbergen SC (2006). Differential direct effects of cyclo-oxygenase-1/2 inhibition on proteoglycan turnover of human osteoarthritic cartilage: An in vitro study. Arthrit. Res. Ther..

[48] El Hajjaji H (2003). Celecoxib has a positive effect on the overall metabolism of hyaluronan and proteoglycans in human osteoarthritic cartilage. J. Rheumatol..

[49] Mastbergen SC (2006). Inhibition of COX-2 by celecoxib in the canine groove model of osteoarthritis. Rheumatol. (Oxf.).

[50] Reijman M (2005). Is there an association between the use of different types of nonsteroidal antiinflammatory drugs and radiologic progression of osteoarthritis? The Rotterdam Study. Arthrit. Rheum..

[51] Simic M (2021). Clinical risk factors associated with radiographic osteoarthritis progression among people with knee pain: A longitudinal study. Arthrit. Res. Ther..

[52] Perry TA (2021). Association between current medication use and progression of radiographic knee osteoarthritis: Data from the osteoarthritis initiative. Rheumatol. (Oxf.).

[53] Cui B (2022). Effects of medications on incidence and risk of knee and hip joint replacement in patients with osteoarthritis: A systematic review and meta-analysis. Adv. Rheumatol..

